# Investigation of side effects to treatment and cause of death in 63 Scandinavian dogs suffering from meningoencephalitis of unknown origin: a retrospective study

**DOI:** 10.1186/s13028-023-00709-7

**Published:** 2023-10-19

**Authors:** Pernille Lindholm Heidemann, Bolette Erhald, Bodil Cathrine Koch, Hanne Gredal

**Affiliations:** 1 Department of Veterinary Clinical Sciences, University of Copenhagen, Dyrlaegevej 16, Frederiksberg, 1870 Denmark; 2 Evidensia Södra Djursjukhuset Kungens Kurva, Månskärsvägen 13, 141 75 Kungens, Kurva, Sweden; 3 Fredrikstad Dyrehospital (Fredrikstad Small Animal Hospital), Wilbergjordet 2, Fredrikstad, 1605 Norway

**Keywords:** Canine, Ciclosporin, Corticosteroid, Inflammatory CNS disease, MUE, MUO

## Abstract

**Background:**

Meningoencephalitis of unknown origin is a common cause of severe neurological disease in dogs. The term covers a heterogeneous group of noninfectious inflammatory diseases, with immune dysregulation widely accepted as the underlying disease mechanism. Current treatment consists of immunosuppression, with corticosteroids being the mainstay of virtually all treatment regimens. However, side effects of corticosteroids can be severe, and might be the cause of death in some patients. This retrospective, multi-centric study aimed at describing a population of Scandinavian dogs with meningoencephalitis of unknown origin in regards to reported side effects and cause of death, and to highlight possible differences in survival, when comparing corticosteroid monotherapy with other treatment regimens.

**Results:**

Within the 5-year study period, 63 dogs were included. Of these, 35 (55.6%) died or were euthanized during the study period. Median survival time from time of diagnosis based on Kaplan-Meier curves for the overall population was 714 days (equivalent to around 25 months, range 0-1678 days). There was no statistically significant difference *(*P = 0.31) in survival between dogs treated with corticosteroid monotherapy (n = 26, median survival time 716 days, equivalent to around 25 months, range 5–911 days), dogs receiving a combination of corticosteroids and ciclosporin (n = 15, median survival time 916 days, equivalent to around 31 months, range 35–1678 days), and dogs receiving corticosteroids combined with either cytosine arabinoside, leflunomide, or a combination of 2 or more add-on drugs (n = 13, median survival time 1186 days, equivalent to around 40 months, range 121–1640 days). Side effects were registered for 47/63 dogs. Polyphagia (n = 37/47), polyuria/polydipsia (n = 37/47), diarrhea (n = 29/47) and lethargy (n = 28/47) were most frequently reported. The most common cause for euthanasia was relapse (n = 15/35, 42.9%), followed by insufficient or lack of treatment response (n = 9, 25.7%). Side effects were the direct cause of euthanasia in 2/35 dogs (5.7%).

**Conclusions:**

A large proportion of dogs in the overall population were euthanized due to relapse, emphasizing a need for treatment regimens aimed at specifically preventing relapse for an improved long-term survival. Side effects in dogs receiving corticosteroid monotherapy were rarely a direct cause of death, but were reported for all dogs. No statistically significant difference in survival was found when corticosteroid monotherapy was compared to other treatment regimens.

## Background

Meningoencephalitis of unknown origin (MUO) is an “umbrella” term covering different histopathological subtypes of noninfectious inflammatory central nervous system (CNS) diseases affecting dogs, including necrotizing meningoencephalitis (NME), necrotizing leucoencephalitis (NLE) and granulomatous meningoencephalitis (GME). However, as a histopathological diagnosis is rarely reached ante mortem, the term MUO is commonly applied in a clinical setting [1]. The pathogenesis is not fully understood, but it is currently deemed most likely to be caused by an inappropriate immune response [2, 3]. This claim is supported by the fact that infectious agents remain unidentified, despite several methodologically different approaches [4–9], and that treatment with immunosuppressive drugs, such as corticosteroids, halters, or reverses disease progression [10]. Furthermore, the presence of neuronal autoantibodies has been demonstrated in some dogs with MUO [11–13].

Different treatment protocols have been suggested; collectively, the purpose is suppression of the immune system, thereby suppressing the improper immune response causing either the formation of perivascular cuffs and a mixed lymphoid inflammatory process (GME) or necrosis and infiltration of lymphocytes, plasma cells and monocytes (NLE and NME) [3, 14]. Monotherapy with corticosteroids (CS) [15, 16], as well as CS in combination with other immunomodulatory therapy such as ciclosporin (Ci) [17], mycophenolate mofetil (MMF) [18], cytosine arabinoside (CA) [19], lomustine (Lo) [20], leflunomide (Le) [21] and azathioprine [22] have been investigated in different studies. Comparison between studies has, however, proven troublesome due to different study designs, inclusion and exclusion criteria, and due to the fact that studies investigating treatment protocols are usually performed retrospectively due to ethical considerations related to patient care [22, 23]. Corticosteroids alone or in combination with other drugs are currently the mainstay of treatment for dogs suffering from MUO [24–26]. However, CS treatment can lead to side effects with both short- and long-term treatment, which in severe cases can be life-threatening [27–32]. In humans, long-term CS treatment has been shown to lead to depression, and to affect multiple organ systems, and may lead to osteoporosis, diabetes mellitus, gastrointestinal bleeding, pancreatitis, hypertension, heart failure, delayed wound healing, glaucoma, predisposition to infection and growth suppression [27–29]. In dogs treated with CS, side effects such as polyuria and polydipsia (PU/PD), polyphagia, weight gain, sleeping more than usual, ‘pot belly’ appearance, gastrointestinal disturbances, thinning of hair coat, dermatitis, hepatomegaly and urinary tract infections have been reported [27, 30, 31]. Gastrointestinal ulcers, a potential life-threatening side effect, have also been described in relation to corticosteroid treatment [32]. Due to the many side effects of CS, protocols aiming at reducing the treatment time are therefore desirable. The claim that no optimal treatment for MUO has yet been identified is illustrated by the large amount of different combination treatment regimens examined in the literature [17–22].

Treatment response to CS in dogs suffering from MUO may vary [26]. Even though the efficacy of CS monotherapy has been investigated [16, 33], previous publications have not looked at side effects and a possible association with mortality in dogs with MUO. In a study investigating quality of life and side effects in dogs suffering from SRMA, severity of side effects correlated with doses of CS [31]. This underlines the importance of investigating dogs with MUO specifically in relation to side effects, since the reported starting doses of prednisolone for MUO range from 2 mg/kg daily to as high as 5 mg/kg daily [16, 20, 33].

The aim of this study was to investigate side effects to treatment, survival and cause of death for a population of Scandinavian dogs diagnosed with MUO. This study further aimed to compare CS monotherapy with combination treatment protocols in regards to survival.

## Methods

### Study setting

This retrospective cohort multicenter study included medical records from dogs presented at the University Hospital for Companion Animals (UHCA), University of Copenhagen, Denmark, and Fredrikstad Small Animal Hospital (FSAH), Fredrikstad, Norway. Initially, medical records from January 2016 to November 2021 from dogs where MUO was considered a differential diagnosis were reviewed, and inclusion and exclusion criteria were then applied.

### Inclusion and exclusion criteria

Inclusion criteria were based on the guidelines proposed by Granger et al. [34] with the following modifications; dogs with chronic NLE/NME were included, and dogs with normal computed tomography (CT) or magnetic resonance imaging (MRI) and cerebrospinal fluid (CSF) analysis were included if MUO remained the most likely diagnosis based on the clinical findings, a positive response to treatment and the exclusion of other causes, as normal findings on both CSF analysis and advanced imaging have been described in dogs with a histopathologically confirmed diagnosis covered by MUO [19, 34]. Dogs in which CSF sampling was contraindicated were also included, if findings on MRI were compatible with MUO. This was done to avoid excluding patients with severe intracranial changes constituting a risk at CSF tap, and thus any selection bias towards a more favorable prognosis. A presumptive diagnosis of MUO was reached based on medical history, clinical and neurological examination, routine hematology and biochemistry, a standard CSF analysis, and either CT or MRI of the brain. A post-mortem histopathological diagnosis of GME, NME or NLE was considered a definite diagnosis. A complete medical record and signalment were further needed for inclusion. For dogs that survived the first 72 h from diagnosis, information from at least one follow-up visit had to be available for inclusion in the part of the study investigating side effects to treatment. Dogs that died within the first 72 h from diagnosis were excluded from treatment subgroups, but not from the overall survival analysis.

In regards to imaging, abnormal findings on MRI examinations, if any, had to be consistent with inflammatory disease (i.e. hyperintense on T2 and FLAIR weighted images, with isointense or hypointense lesions on T1 weighted images) [35, 36]. Even though it is not always possible to differentiate inflammation from other diseases based on MRI [36], some findings can support the diagnosis of inflammation, and of MUO in particular, and so these specific characteristics were taken into account. In general, meningoencephalitis often have multifocal lesions of irregular shape and ill-defined margins. Contrast enhancement can be variable and can include meninges. Specifically, for GME, focal lesions, as well as diffuse and multifocal lesions can be observed, with focal lesions sometimes indistinguishable from neoplasia. In cases where neoplasia and GME could not be distinguished on MRI, a diagnosis was based on CSF results compatible with inflammatory disease as well as a positive response to treatment [37].

Dogs, in which only CT was available were included with imaging changes considered compatible with MUO, i.e., ill-defined hypo-attenuating lesions with/without variable and ill-defined contrast enhancement [38]. However, acknowledging the low sensitivity of CT in the detection of inflammatory CNS disease [34], dogs with a normal CT scan, but CSF results compatible with inflammatory disease and a positive response to treatment were also included. Dogs in which other pathological causes, e.g. neoplasia, were identified on CT, were excluded from the study.

Dogs were excluded if there were insufficient medical records, other neurological diseases identified explaining clinical signs, if histopathology was non-diagnostic, or if they suffered from severe concurrent systemic disease. Dogs diagnosed with meningomyelitis only were also excluded.

All information from patient records regarding signalment, clinical and neurological findings, results from paraclinical examinations, registered side effects, treatment response and cause of death were noted. For cause of death, dogs were allocated to one of the following: ‘Spontaneous death’, ‘Euthanized at diagnosis’ (either by owner request or recommended by the responsible veterinarian), ‘No treatment response’ (when treatment were attempted, and no improvement were registered), ‘Relapse’ (when an initial treatment response was seen, followed by a relapse either in relation to tapering or after tapering. Euthanasia had to be performed in relation to the registered relapse for dogs to be categorized in this group), ‘Side effects’ (when side effects and quality of life according to the patient records resulted in euthanasia), ‘Possible side effects’ (when cause of death may indirectly be attributed to treatment), or ‘Other’ (including death with no obvious relation to disease or treatment). Changes in weight were calculated based on weight at initial presentation and the last registered weight in the patient record. Changes in body weight were investigated in relation to treatment (data missing in 1/47). For dogs where no time of death was registered, owner contact for a clinical status on their dog was attempted.

Included dogs were further allocated to one of the following subgroups: Monotherapy with CS, therapy with CS + Ci, therapy with CS + CA, therapy with CS + at least two add-ons (CS + 2), and therapy with CS + Le. Dogs that died or were euthanized within the first 72 h from diagnosis were allocated to a separate group termed ‘early death’. This cut-off was set based on previous studies [19], showing a high mortality rate specifically in this time period. Inclusion of these dogs in a treatment group was therefore likely to bias the monotherapy group negatively, as all dogs initially were started on CS, and death before add-on treatment could be initiated would lead to inclusion in the monotherapy group.

### Paraclinical investigations

In order for dogs to be included, diagnostics to rule out other diseases as the cause of clinical signs needed to be performed, as deemed by the responsible clinician.

CSF analysis was either performed in-house (UHCA) or sent to an external laboratory (IDEXX Reference Laboratories GmbH, Leipzig, Germany (FSAH)). Analysis included a macroscopic evaluation, protein concentration measurement, manual red blood and total nucleated cell counts, and a differential cell count. Protein content was either measured semi-quantitatively using a urine multi-dipstick (Siemens Multistix® 10 SG, UHCA) or quantitatively (IDEXX Reference Laboratories GmbH, Leipzig, Germany (FSAH)). At UHCA, MRI examinations were performed using a 0.2 Tesla magnet (Esaote, VetMR) or a 1.5 Tesla magnet (Phillips). At FSAH, a 1.5 Tesla magnet (GE Healthcare) was used for all examinations. For all MRI examinations, a gadolinium-based contrast was given at a dosage of 0.1 mmol/kg IV. CT examinations were performed using either Siemens Somatom Emotion single-slice helical CT-scanner (UHCA) or a GE Healthcare Brivo CT 385 16-slice helical CT-scanner (FSAH), and an iohexol-based contrast was used at a dosage of 300–600 mg/kg IV.

### Statistical analysis

Data regarding signalment, initial neurological examination, paraclinical parameters, CSF analysis, advanced imaging, side effects, and cause of death were presented descriptively. Median, mean and range were calculated when applicable for all data. Side effects were described for the overall population, and for treatment subgroups. Median survival time from the time of diagnosis (MST) based on Kaplan-Meier survival curves were evaluated for the overall population, for the two largest treatment subgroups separately (CS and CS + Ci), and the three smaller treatment subgroups (CS + CA, CS + Le, CS + 2) combined. Even though the combination of different treatment groups did not allow for a differentiation between their effects on survival, an overall survival could be calculated, and compared to CS, as well as CS + Ci. The three subgroups were compared using Gehan-Breslow-Wilcoxon test with the statistical significance level set at P < 0.05 (GraphPad Prism 9, GraphPad Software, San Diego, USA). Since survivors are censored from the Kaplan-Meier survival curve steps, dogs that were lost to follow-up or still alive at the end of the study period were visualized with thick marks directly on the curve. To give an overview of which dogs fell into which group, and to allow for comparison to other studies, a summary of the population at different time intervals was made (1 month, 3 months and 12 months).

## Results

**Fig. 1:**
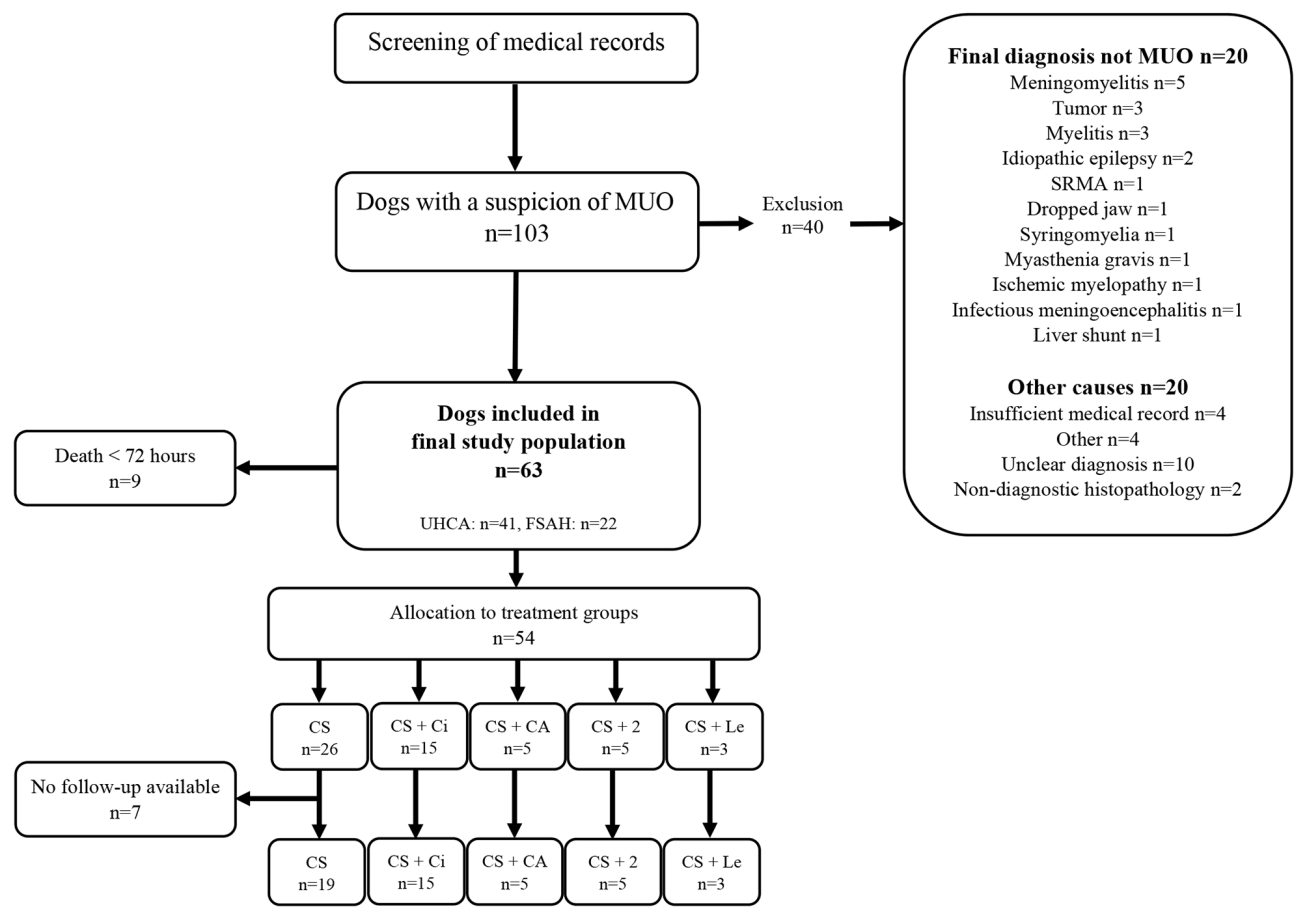
Overview of study population. Set up of the retrospective multicenter study of MUO with inclusion and exclusion of dogs based on medical record reviews. A total of 63 dogs were included in the final population. Abbreviations: +2 = two or more add-on medications, CA = cytosine arabinoside, Ci = ciclosporin, CS = corticosteroids, FSAH = Fredrikstad Small Animal Hospital, Le = leflunomide, MUO = meningoencephalitis of unknown origin, SRMA = steroid responsive meningitis arteritis, UHCA = University Hospital for Companion Animals

The initial investigation of patient records yielded 103 dogs with MUO as a possible differential diagnosis. Of these, 20 were diagnosed with other diseases as the cause of clinical signs and thus excluded, and 20 were excluded due to other causes, leaving 63 dogs in the study population (see Figs. 1), 41 from UHCA, and 22 from FSAH. For two of the included dogs, advanced imaging (either CT or MRI) was not performed. One dog died before imaging and collection of CSF were performed, but was diagnosed with NME on histopathology. The other was a young pug dog (10 months old) with seizures and signs of multifocal cerebral involvement on neurological examination, as well as a moderate mononuclear pleocytosis (TNCC 95/µL) on CSF analysis. This dog was included due to the lack of other likely differential diagnoses.

The majority of dogs in the study population weighed < 15 kg (n = 53/63, 84.1%). Chihuahuas (n = 12), Pugs (n = 9) and Yorkshire terriers (n = 5) were the most common breeds. Mean age at presentation was 56.6 months (median 54.0 months, range 7–157 months). A summary of the signalment for the study population can be seen in Table 1.

**Table 1:** Signalment for all dogs included in the study (n = 63)

Breeds (n)	Chihuahua (12), Pug (9), Yorkshire terrier (5), Small mixed breed (5), Bichon Havanais (4), Coton de Tulear (3), French Bulldog (3), Maltese (2), Welsh Springer Spaniel (2), Boston Terrier (2), Basset Hound (1), Border Collie (1), Boxer (1), Bichon Frisé (1), Cairn Terrier (1), Dobermann (1), Griffon Bruxellois (1), Medium Poodle (1), Nova Scotia Duck Tolling Retriever (1), Papillon (1), Petit Brabancon (1), Pomeranian (1), Prazsky Krysarik (1), Puli (1), Weimaraner (1), Whippet (1)					
Age (months)	**Mean**: **Median**:	56.6 54.0	**Range**	7-157		
Weight (kg)	**Mean**: **Median**:	8.3 5.8	**Range**	1.6–42	**< 15**: **≥ 15**:	53 (84.1%) 10 (15.9%)
Gender	**Female**: **Male**:	36 (57.1%) 27 (42.9%)	**Neutered**: **Intact**:	11 (17.5%) 52 (82.5%)		

The time from onset of clinical signs until the first neurological examination varied greatly. 37/63 dogs (58.7%) were presented for examination within seven days of clinical onset; 8/63 dogs (12.7%) between 8 and 14 days, and 9/63 dogs (14.3%) between 15 and 30 days of clinical onset. 9/63 dogs (14.3%) had had clinical signs for more than 30 days at the time of presentation. For 2 of these dogs (3.2%), clinical signs had been present for more than six months.

### Neurological examination

On initial neurological examination, 15/63 dogs (23.8%) were described as alert. 40/63 dogs (63.5%) were deemed depressed, 1/63 dogs (1.6%) was stuporous, and 3/63 dogs (4.8%) presented in a comatose state. The mental state of the remaining 4/63 dogs (6.3%) could not be evaluated, as they presented in either status epilepticus (3/63 dogs, 4.8%) or were in a medically induced sedated state (1/63 dogs, 1.6%). Notably, a history of seizure activity was given in more than a third of the included dogs (27/63 dogs, 42.9%).

40/63 dogs (63.5%) presented with abnormal movement, with 37/63 (58.7%) presenting with ataxia, and 2/63 (3.2%) presenting with paresis/plegia. Proprioceptive placing reactions were affected on one or more legs in 39/63 dogs (61.9%). Of these 39 dogs, 23/63 (36.5%) had proprioceptive placing deficits on one side. On cranial nerve examination, the most commonly reported finding was reduced menace response, which was noted in 31/63 dogs (49.2%). Findings on neurological examinations are summarized in Table 2.

**Table 2:** Neurological findings on initial presentation (n = 63)

			n	%
Abnormal mentation			44	69.8
	Depressed		40	63.5
	Stuporous		1	1.6
	Comatose		3	4.8
	N/A		4	6.2
Deviating behavior			32	50.8
	Abnormal (not specified)		7	11.1
	Circling		23	36.5
	Hemineglect		2	3.2
Seizures			27	42.9
	Generalized seizures*		14	22.2
		Cluster seizures	6	9.5
		Status epilepticus	2	3.2
	Focal seizures		5	7.9
Abnormal movement			40	63.5
	Paresis/plegia		2	3.2
		Tetraparesis	1	1.6
		Hemiparesis	1	1.6
	Ataxia		37	58.7
		Generalized ataxia	26	41.3
		Ataxia affecting pelvic limbs	7	11.1
		Ataxia with hypermetria	4	6.4
	Leaning towards one side		1	1.6
Abnormal body posture*			26	41.3
	Pleurotothonus		3	4.8
	Head turn		12	19.0
	Head tilt		17	27.0
	Both head turn and head tilt		4	6.3
Proprioceptive placing deficits			39	61.9
	One limb		7	11.1
	One side		23	36.5
	Pelvic limbs		3	4.8
	Thoracic limbs		1	1.6
	All limbs		5	7.9
Abnormal findings on cranial nerve examination*			41	65.1
	Strabismus		15	23.8
		Positional strabismus	4	6.3
	Abnormal menace response		31	49.2
		Abnormal unilateral	16	25.4
		Abnormal bilateral	15	23.8
	Pathological nystagmus		7	11.1
		Rotating	1	1.6
		Vertical	4	6.3
		Horizontal	2	3.2
	Abnormal nasal sensation		13	20.6
		Abnormal unilateral	10	15.9
		Abnormal bilateral	3	4.8
Spinal pain			20	31.7
	Cervical		17	27.0
	Thoracic		1	1.6
	Not localized		2	3.2
Localization based on neurological examination				
	Multifocal		34	54.0
	Cerebrum		17	27.0
	Brain stem		5	7.9
	No localization specified		7	11.1

*Overview of findings at initial examination of 63 dogs with a tentative diagnosis of meningoencephalitis of unknown origin.* N/A = Dogs that presented either in seizures or sedated. Mental status could therefore not be evaluated at presentation. *Dogs may be counted in more than one of the below mentioned abnormalities.

A clinical neurolocalization was noted for 56/63 dogs (88.9%), with 34/63 dogs (54.0%) being characterized as having a multifocal localization. 22/63 dogs (34.9%) had signs compatible with a focal neurolocalization: Cerebrum (n = 17/63, 27.0%) or brain stem (n = 5/63, 7.9%). For 7/63 dogs (11.1%), localization was not specified.

### Blood tests and infectious diseases

For all dogs, as a minimum, routine hematology and blood biochemistry were performed. Samples were either analyzed at place of inclusion, at the referring veterinarian, or at an external laboratory. For 48/63 dogs, analysis of canine C-reactive protein (CRP) was available. 12/48 dogs (25.0%) had a CRP above the local reference range (upper reference ranging from 10 to 30 mg/L, depending on place of analyses). Tests for infectious diseases were performed at the clinician’s discretion. The most frequently performed test (16/63 dogs) was for antibodies against *Angiostrongylus vasorum* (Angio Detect Test, Idexx, Netherlands). Other performed tests included investigation for antibodies against *Anaplasma phagocytophilum* and/or *platys* (not differentiated by the test), *Borrelia burgdorferi*, *Ehrlichia canis*, *Ehrlichia ewingii*, and *Dirofilaria immitis*, which were performed in 5/63 dogs (SNAP 4Dx plus, Idexx, Netherlands), investigation of serum antibodies against Leptospira (1/63 dogs), PCR for tick borne encephalitis virus and/or the agent causing Lyme disease (2/63), and serum antibody measurements for *Toxoplasma gondii* (IgG and IgM) and/or *Neospora caninum* (2/63). All tests for infectious diseases were negative, apart from a snaptest (SNAP 4Dx Plus Test, Idexx, Netherlands) performed in one dog, which was positive for *Anaplasma* spp. This test result was not followed up with PCR analysis, as *Anaplasma* spp. was deemed unlikely as the cause of the clinical signs, and multifocal lesions explaining signs were identified on MRI examination of the brain. The dog responded to treatment with CS + Ci and lived 24 months after initial diagnosis.

### Advanced imaging

MRI examination was performed in 34 dogs. In 3/34 (8.8%) the MRI study was deemed normal. For the dogs where MRI was available for review, and deemed abnormal (30 dogs), all had hyperintense lesions on both T2-weighted and FLAIR images. In 26/30 dogs (86.7%) enhancement of identified lesion(s) or a patchy diffuse enhancement were noted post contrast. Multifocal lesions were most commonly reported (14/30, 46.7%), followed by diffuse lesions (11/30, 36.7%) and focal lesions (5/30, 16.7%). Lesions were most often localized to cerebrum (25/30 dogs, 83.3%), followed by lesions in the brain stem (3/30 dogs, 10.0%), spinal cord (3/30 dogs, 10.0%), thalamus (2/30 dogs, 6.7%) and cerebellum (1/30 dogs, 3.3%). In 5/30 dogs (16.6%), imaging findings consistent with cerebellar herniation were reported, with one of these dogs also having findings consistent with caudal transtentorial herniation. In 2/30 dogs (6.7%), signs of elevated intracranial pressure were noted. In one dog, MRI was performed at another animal hospital. Images were therefore not available for review, but the imaging report specified two findings; one lesion in the caudal medulla oblongata, and one lesion related to the right olfactory recess. Both lesions were consistent with an inflammatory process with necrotic changes.

CT examinations were available for 27 dogs. In 6/27 (22.2%) dogs, either multifocal lesions (5/27, 18.5%) or focal lesions (1/27, 3.7%) were noted. In all six dogs, contrast uptake was noted, and in two, a rim-like contrast enhancement was seen. In 3/27 dogs (11.1%), lesions could not be classified as focal or multifocal, but mass effect was noted. In 19/27 dogs (70.4%), no signs of inflammatory disease were noted. Signs of cerebellar herniation were reported in 1/27 dogs (3.7%).

### Cerebrospinal fluid analysis

Results of a complete or partial CSF analysis were available for 45/63 dogs. Reasons for missing analyses were clinical contraindications for CSF tap (n = 10), marked blood contamination (n = 4), death or euthanasia before CSF tap could be performed (n = 1), and causes not specified (n = 3).

An exact total nucleated cell count (TNCC) was available for 30/45 dogs. For five other dogs, pleocytosis was noted, but the TNCC was not specified. The median TNCC was 94 cells/µL (range 1-4000 cells/µL, mean 394 cells/µL). The TNCC was > 5 cells/µL in 32/35 dogs (91.4%). For 3/35 dogs (8.6%), TNCC was normal (TNCC ≤ 5/µL).

Cytological evaluation was available for 45 dogs. Of these, the three dogs that had a normal TNCC also had a normal cytological evaluation. The remaining 42/45 dogs (93.3%) had a pleocytosis. 31/45 dogs had a pleocytosis with predominance of monocytes; of these, 14/45 dogs (31.1%) had a predomination of small mononuclear cells, 5/45 (11.1%) had a predominance of large mononuclear cells, and 12/45 (26.7%) had a pleocytosis with almost equal amounts (40–60% of each) of small and large mononuclear cells. A mixed pleocytosis, with a neutrophil count ≥ 25%, was reported in 6/45 dogs (13.3%). For five dogs, cytological evaluation was reported as abnormal without further specification.

Total protein concentration measurement was available for 34 dogs. For 8/34 dogs (23.5%), the protein count was less than 30 mg/dL, and therefore deemed normal. For 26/34 dogs (76.5%), total protein concentration was above 30 mg/dL. Of these, 12/34 dogs (35.3%) had a protein count from > 30–100 mg/dL, 10/34 dogs (29.4%) had an abnormal protein concentration between > 100–300 mg/dL, 3/34 dogs (8.8%) had a total protein count between > 300–2000 mg/dL, and 1/34 dog (2.9%) had a protein count > 2000 mg/dL. This particular dog was the same dog that had the highest TNCC overall in the study (4000 cells/µL).

### Treatment and side effects

Treatment was registered as follows: CS: n = 26, CS + Ci: n = 15, CS + CA: n = 5, CS + 2: n = 5 and CS + Le: n = 3. Of these, at least one follow-up visit was registered for all but seven dogs in the CS monotherapy group, leaving 19 dogs in this group for assessment of side effects. A total of 16 dogs were excluded from investigation of side effects, due to either early death (n = 9) or a lack of follow-up visits registered (n = 7), leaving 47 dogs in total. For an overview of medications given initially, see Table 3.

**Table 3:** Overview of treatment regimens

Treatment Group	Dogs (n)	Median initial dose	Mean initial dose	Range
**CS**	26			
Dexamethasone	20	0.5 mg/kg/day	0.5 mg/kg/day	0.2–1.15 mg/kg/day
Prednisolone	26	2.2 mg/kg/day	2.4 mg/kg/day	1.73-4 mg/kg/day
**CS + Ci**	15			
Dexamethasone	13	0.5 mg/kg/day	0.54 mg/kg/day	0.4–0.98 mg/kg/day
Prednisolone	15	2.5 mg/kg/day	2.63 mg/kg/day	2–4 mg/kg/day
Ciclosporin	15	6 mg/kg/day	6.4 mg/kg/day	5.43-12 mg/kg/day
**CS + CA**	5			
Dexamethasone	4	0.44 mg/kg/day	0.47 mg/kg/day	0.4–0.6 mg/kg/day
Prednisolone	5	1.9 mg/kg/day	2.11 mg/kg/day	1.73-3 mg/kg/day
Cytosine arabinoside	5	200 mg/m^2^	175 mg/m^2^	100–200 mg/m^2^
**CS + 2**	5			
Dexamethasone	4	0.5 mg/kg/day	0.625 mg/kg/day	0.5-1 mg/kg/day
Prednisolone	5	2 mg/kg/day	2.0 mg/kg/day	1.78–2.3 mg/kg/day
Ciclosporin	2	6 mg/kg/day	6 mg/kg/day	6 mg/kg/day
Cytosine arabinoside	3	100 mg/m^2^	133 mg/m^2^	100–200 mg/m^2^
Leflunomide	1	2 mg/kg	2 mg/kg	2 mg/kg
Lomustine	1	30 mg/m^2^	30 mg/m^2^	30 mg/m^2^
**CS + Le**	3			
Dexamethasone	1	0.5 mg/kg/day	0.5 mg/kg/day	0.5 mg/kg/day
Prednisolone	3	2.29 mg/kg/day	2 mg/kg/day	2–4 mg/kg/day
Leflunomide	3	1.04 mg/kg/day	1.04 mg/kg/day	0.58–1.5 mg/kg/day

*Overview of different treatment regimens and distribution of included dogs surviving > 72 h from time of diagnosis (n = 54) to different treatment groups* *. CA = cytosine arabinoside, Ci = ciclosporin, CS = corticosteroids, Le = leflunomide, + 2 = more than 2 add-on drugs. Treatment with cytosine arabinoside was given initially as IV-infusion, followed by subcutaneous injections every 12 h for 48 h every 3–4 weeks. Dogs receiving dexamethasone received it before starting oral treatment with prednisolone.*

The most commonly reported side effects were polyphagia (37/47, 78.7%) and PU/PD (37/47, 78.7%). Gastrointestinal signs such as diarrhea (29/47, 61.7%) and vomiting (17/47, 36.2%), were also commonly noted. A weight gain of more than 10% was seen in 24/46 dogs (52.2%), whereas weight loss of more than 10% was only seen in 3/46 dogs (6.5%). Only slight differences in side effects were seen between treatment subgroups. 1/5 dogs in the CS + 2 group developed gingival hyperplasia. 5/47 dogs developed corneal ulcers during treatment, two in the CS group, two in the CS + Ci group and one in the CS + 2-group. Side effects are summarized in Table 4.

**Table 4:** Side effects noted during treatment

	All dogs (n = 47) n (%)	CS (n = 19) n (%)	CS + Ci (n = 15) n (%)	CS + CA (n = 5) n (%)	CS + 2 (n = 5) n (%)	CS + Le (n = 3) n (%)
Polyphagia	37 (78.7)	15 (78.9)	11 (73.3)	4 (80.0)	4 (80.0)	3 (100)
PU/PD	37 (78.7)	13 (68.4)	14 (93.3)	3 (60.0)	4 (80.0)	3 (100)
Diarrhea	29 (61.7)	13 (68.4)	9 (60.0)	3 (60.0)	4 (80.0)	
Lethargic	28 (59.6)	12 (63.2)	8 (53.3)	3 (60.0)	3 (60.0)	2 (66.7)
≥ 10% weight gain	24 (52.2)^III^	9 (47.4)	8 (57.1)^III^	3 (60.0)	3 (60.0)	1 (33.3)
Behavioral change	20 (42.6)	9 (47.4)	8 (53.3)	1 (20.0)	1 (20.0)	1 (33.3)
Vomiting	17 (36.2)	10 (52.6)	5 (33.3)		2 (40.0)	
Muscle atrophy	16 (34.0)	10 (52.6)	5 (33.3)			1 (33.3)
Pendulating abdomen	14 (29.8)	3 (15.8)	7 (46.7)	1 (20.0)	2 (40.)	1 (33.3)
Infections^I^	11 (23.4)	6 (31.6)	3 (20.0)	1 (20.0)		1 (33.3)
Poor coat quality	11 (23.4)	4 (21.1)	5 (33.3)		1 (20.0	1 (33.3)
Panting	10 (21.3)	5 (26.3)	2 (13.3)	1 (20.0)	1 (20.0)	1 (33.3)
Exercise intolerance	8 (17.0)	3 (15.8)	4 (26.7)	1 (20.0)		
Corneal ulcer	5 (10.6)	2 (10.5)	2 (13.3)		1 (20.0)	
Anorexia	4 (8.5)	3 (15.8)	1 (6.7)			
≥ 10% weight loss	3 (6.5)^III^	1 (5.3)	2 (14.3)^III^			
Other^II^	6 (12.8)	1 (5.3)	3 (20.0)	1 (20.0)	1 (20.0)	

*Overview of side effects registered for dogs with at least one registered follow-up visit, as well as the distribution between different treatment groups (n = 47).* ^*I*^ *Of the dogs with infections registered during treatment, six had infections primarily related to the skin. Four dogs presented with either pneumonia (1), sepsis and peritonitis (1), bacterial cystitis (1) or an eye infection (1). One dog presented several times during treatment with infections, which included a suspected discospondylitis, osteomyelitis in one toe, skin infections, as well as a tooth abscess.* ^*II*^ *Other: CS: Crust-like skin lesions, CS + Ci: Calcinosis cutis, tooth loss, thrombi formation, CA: Tooth loss, CS + 2: Gingival hyperplasia.* ^*III*^ *Data regarding weight changes were missing for 1 dog. +2 = 2 or more add-ons, CA = cytosine arabinoside, All = all dogs with reported side effects, Ci = ciclosporin, CS = corticosteroids, Le = Leflunomide, PU/PD = Polyuria/Polydipsia.*

### Survival and cause of death

**Fig. 2:**
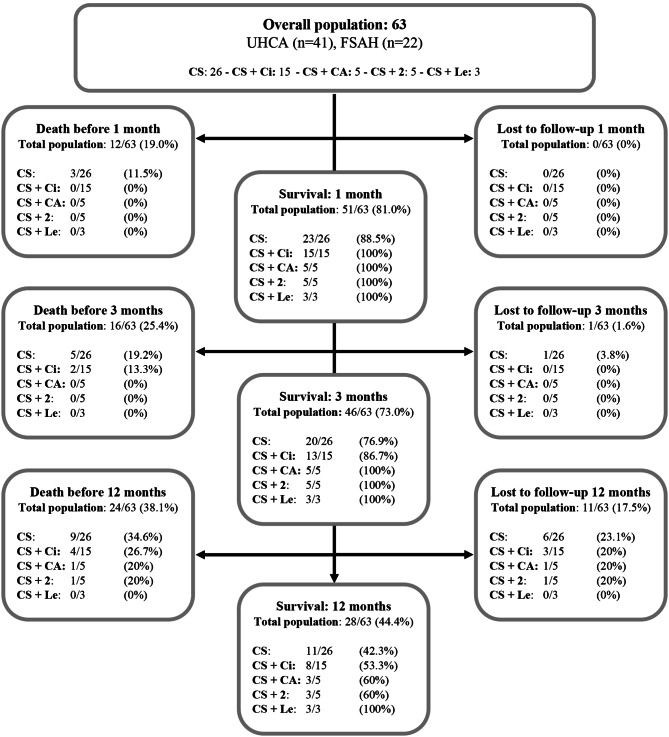
Overview of death, survival and dogs lost to follow-up. Overview of survival, death and number of dogs where follow-up were too short at different time intervals. CA = cytosine arabinoside, Ci = ciclosporin, CS = corticosteroids, FSAH = Fredrikstad Small Animal Hospital, Le = Leflonumide, UHCA = University Hospital for Companion Animals, + 2 = at least two add-ons

Overall, 35/63 dogs (55.6%) died within the 5-year study period. At the one-year mark, 38.1% (24/63 dogs) were dead, 44.4% (28/63 dogs) were alive, and 17.5% (11/63 dogs) were lost to follow-up. In the CS group, 13 dogs (50.0%) died during the study period, and in the CS + Ci-group, 8 dogs (53.3%) died. See Fig. 2 for an overview of the study population at the 1 month, 3 months and 12 months mark.

**Fig. 3:**
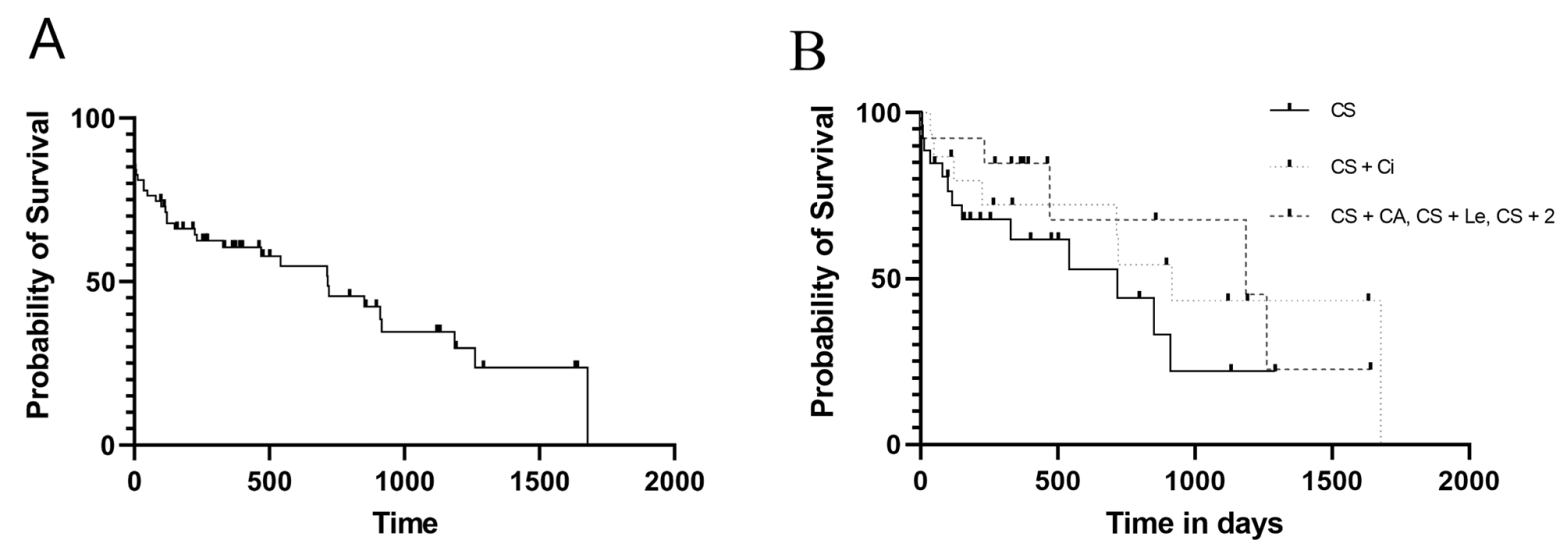
Kaplan-Meier survival curves for the overall population (3 A) and subgroups (3B). Thick marks represent survivors where death did not occur during the study period, or the dog was lost to follow-up. Time in days, *probability* in %. **3A**: Overall population. MST = 714 days (range 0-1678 days). **3B**: Subgroups. MST for CS = 716 days (range 5-911 days), MST for CS + Ci = 916 days (range 35-1678 days), MST for CS + CA, CS + Le, CS + 2 = 1186 days (121–1640 days). CS = corticosteroids, Ci = ciclosporin, CA = cytosine arabinoside, Le = leflunomide, MST = median survival time, + 2 = two or more add-on drugs

Kaplan-Meier curves for the overall population (n = 63), as well as the three separate subgroups (CS n = 26; CS + Ci n = 15; CS + CA, CS + Le, CS + 2 n = 13), can be seen in Fig. 3. Median survival time based on the Kaplan-Meier survival curve was 714 days (range 0–1678 days) for the overall population, 716 days (range 5–911 days) for the CS group, 916 days (range 35–1678 days) for the CS + Ci group, and 1186 days (range 121–1640 days) for the combination group. There was no statistically significant difference in survival curves between the three groups (P = 0.31).

The most frequently reported cause of death in general was relapse of disease (15/35, 42.9%), followed by a lack of response to treatment (9/35, 25.7%). Of the 35 dogs that died during the study period, 9/35 (25.7%) died or were euthanized within the first 72 h from diagnosis. Of these, five were euthanized due to a lack of initial response to treatment, three were euthanized at diagnosis, and one died spontaneously. Overall, only 2/35 dogs (5.7%) were euthanized due to causes directly related to side effects from medication, one in the CS group, and one in the CS + Le group. For the dog in the CS group, fatigue, vomiting, PU/PD, poor coat quality, muscle atrophy, panting, and a weight loss of 14.3% of initial body weight were described. The dog also further presented with a corneal ulcer and skin wounds with slow healing and in the end developed severe gastrointestinal signs. The dog was euthanized after 99 days of treatment based on the recommendation of the veterinarian responsible for its treatment. For the dog in the CS + Le group, PU/PD, polyphagia and lethargy were reported, and the owner elected euthanasia after a treatment period of 469 days. Cause of death in five dogs was not reported as directly related to MUO or treatment. However, of these, the cause of euthanasia was pancreatitis in one dog, and a gastrointestinal foreign body in the other; both dogs were in the CS group, and euthanasia could potentially be related to treatment with corticosteroids, as both could potentially be secondary to polyphagia - cause of death was therefore classified as possibly related to treatment. Causes of death are summarized in Table 5.

**Table 5:** Cause of death

	Number of deaths		Spontaneous death		Euthanized at diagnosis		No treatment response		Relapse		Side effects		Possible side effects		Other	
	n	%	n	%	n	%	n	%	n	%	n	%	n	%	n	%
All dogs **n = 63**	35	55.6	1	2.9	3	8.6	9	25.7	15	42.9	2	5.7	2	5.7	3	8.6
Early death **n = 9**	9	23.4	1	11.1	3	33.3	5	55.5								
CS **n = 26**	13	50.0					3	23.1	5	38.5	1	7.7	2	15.4	2	15.4
CS + Ci **n = 15**	8	53.3					1	12.5	6	75.0					1	12.5
CS + CA **n = 5**	1	20.0							1	100						
CS + 2 **n = 5**	2	40.0							2	100						
CS + Le **n = 3**	2	66.7							1	50.0	1	50.0				

*Overview of cause of death or euthanasia for the dogs that died during the 5-year study period (n = 35). +2 = 2 or more add-ons, CA = cytosine arabinoside, Ci = ciclosporin, CS = corticosteroids, ‘Early death’ = Dogs that died or were euthanized within the first 72 h from diagnosis, Le = Leflunomide*

## Discussion

This study aimed to investigate cause of death and side effects in a Scandinavian population of dogs with MUO, as well as to compare survival between dogs receiving different treatment regimens. The study population closely resembles those of previous studies with regard to age and weight but included a larger proportion of Chihuahuas and Yorkshire terriers [19, 26], likely due to the geographical localization of the two animal hospitals in areas dominated by apartment-dwelling owners. Dogs presented with a wide variation of neurological signs, mirroring the fact that the umbrella term MUO covers both GME, NME and NLE, known to affect different localizations of the brain [1]. Many of the included dogs presented with multifocal localizations on initial neurological examination (34/63, 54.0%), which is in agreement with previous publications [34, 39]. A significant proportion of the included dogs (27/63, 42.9%) either had a history of recent seizures (focal or generalized), or presented in ongoing seizure activity, indicating forebrain pathology. This is also in line with previous literature in which 37.9–47% presented with seizures as part of their history [39, 40].

The MST in the total study population was 714 days (range 0-1678 days). There was no statistically significant difference when comparing survival for dogs treated with CS monotherapy (MST 716 days, range 5-911), CS + Ci combination therapy (MST 916, range 35-1678), or dogs receiving other combination therapies in the current study population (MST 1186, range 121–1640 days) *(*P = 0.31). However, the limited sample size of all three investigated groups must be kept in mind, and a potential bias towards more severely affected animals in the two subgroups with dogs receiving combination therapy should be considered. In the current study, dogs with more severe clinical signs or an insufficient response to treatment were more likely to receive combination therapy; this is especially true for the dogs included at the UHCA (41/63 dogs), since CS monotherapy in general was the treatment of choice during the five-year study period. On the other hand, dogs receiving combination therapy may be representative of more dedicated owners, as these dogs may need more clinical check-ups and are more costly; this could potentially bias data towards longer survival in this group. A clinical trial with a randomized allocation of dogs to treatment groups with or without add-ons (case control) at the time of diagnosis could be useful. However, this approach may not be ethically acceptable. Alternatively, already existing retrospective raw data could be pooled from multiple locations with sufficient knowledge and diagnostic equipment. This would allow for a larger study population, as well as representation of different treatment regimens since preferred treatment regimens likely differ between different animal hospitals – strict alignment of inclusion and exclusion criteria, as well as standardization of data registration would, however, need to be applied.

Direct comparison to available retrospective studies of MUO survival in regards to treatment regimens is challenging due to differences in study set-ups and inclusion and exclusion criteria. Further, Kaplan-Meier survival curves, which are used in a variety of studies to calculate survival in regards to treatment of MUO [16–18, 20, 22, 23, 25, 26, 33, 41–47], do partially censor study subjects that are still alive at the end of the study period [48]. Even though Kaplan-Meier survival analysis is likely to be the best approach for calculation of survival, disadvantages must be kept in mind. In the current study, cut-off for survival, death or lost to follow-up was made at 1 month, 3 months and 12 months, in order to supplement data that would not be visible on a Kaplan-Meier survival curve, as well as to allow comparison to studies previously made and, in the future, where another approach for calculating MST is used.

In a study by Paušová et al. [15], 182 dogs treated with CS monotherapy had a MST of 570 days (range 2-3540), and a one-year survival of 55.6%. Median survival time was, however, not calculated using Kaplan-Meier survival curves, and even though a 10-year follow-up allowed for exact survival time for most dogs, some long-term survivors were censored from calculations of MST, making direct comparison difficult, as this presumably affects MST negatively. However, the resulting one-year survival in Paušová’s study [15] is longer than the one seen in the current study for the overall population (44.1%), as well as for the CS group (42.3%). However, a substantial number of the dogs in our study were lost to follow-up (17.5% in the overall population and 23.1% in the CS group). One-year survival in our study was similar to Paušová’s study [15] for the CS + Ci group. Around 20% in this group were also lost to follow-up. Survival was higher in the three other combination groups (CS + CA: 60%, CS + 2: 60%, CS + Le: 100%), but small group size needs to be taken into account.

The combination of CS + Ci for treating MUO was investigated in a retrospective study by Brady et al. [47], where a survival of 1345 days (range 38–2044) from diagnosis was reported (n = 40). These results seem very convincing in demonstrating a longer survival than the one shown in the overall population of our study, and when compared to our CS + Ci group. However, in Brady’s study, dogs were only included if they tested negative for infectious diseases at initial presentation or, in the absence of testing (5/40), were still alive one year after diagnosis. This way of inclusion potentially introduces a bias towards a longer survival, and results should be interpreted with caution.

Cytosine arabinoside have been investigated for treating MUO in several studies [19, 41, 43, 45, 46] with results indicating a great potential. In a study by Lowrie et al. [41], a 90% survival (n = 41) was seen at the 3 months mark from diagnosis, if CA was given as a constant rate infusion (CRI) initially, followed by SC injections, and combined with corticosteroids. Dogs that survived the first 3 months, were all alive after 1 year. A lower survival of 44% (n = 39) was seen after 3 months when CA was exclusively given as SC injections in Lowrie et al. [19]. However, none of the dogs that were alive at 3-months died before the 1-year mark. In our study, 73.0% (46/63) were alive after 3 months, but only 44.4% (28/63 dogs) were alive after one year. Survival in Lowrie et al. [41] and Lowrie et al. [19] seem superior to our study, especially if dogs survive the first 3 months. However, continuous treatment with CA can be a challenge, as it needs to be administered at an animal hospital, raising cost when compared to other alternatives. Interestingly, when looking at the challenges related to continuous treatment with CA, Stee et al. [44] compared dogs treated only with CA CRI (n = 42) to a group of dogs (n = 42) receiving both an initial CRI of CA, and subsequent SC treatment. No statistical difference was found between groups, and success rate were almost identical. Since it seems that dogs suffering from MUO can benefit just as much from receiving only the initial CRI infusion, CA could be a viable treatment option in most cases. Only five dogs in our study were treated with CA; due to the small size of this treatment group, statistical evaluation of this group alone was not possible. However, all five dogs were alive at the 3-month mark, as opposed to the CS group (20/26 dogs alive) and CS + Ci group (13/15 alive). In line with current literature, CS + CA seems to be an effective treatment with a good long-term survival overall.

Little is published regarding treatment of MUO with Le. In Gregory et al. [21] Le and its effect on different supposedly immune mediated diseases were investigated. The study included five dogs with either multifocal nonsuppurative encephalitis or meningomyelitis [21]. All five dogs had a good to excellent improvement in neurological status, and two dogs had a partial resolution of cortical lesions on follow-up MRI scans. In regards to side effects, a case series including 14 dogs suffering from immune-mediated polyarthritis, deemed the drug both safe and effective, with only one dog lacking a clinical response to treatment. One dog was reported to have anorexia and vomiting, two dogs had a mild leukopenia, and one dog had a mild thrombocytopenia [49]. In our study, three dogs were treated with Le, and were alive at the one-year mark. However, studies investigating larger populations receiving Le are warranted before any conclusions can be drawn.

Other immunomodulatory drugs that have been investigated in the attempt to treat dogs with MUO includes MMF [18, 23, 42] and azathioprine [22]. None of the dogs included in our study received either of these drugs, but studies investigating their effect may be of interest, and especially the combination of CS and azathioprine, showed promising results in a study by Wong et al. [22], who reported a MST of 1836 days (range 50–2051 days) in 40 dogs with MUO. However, azathioprine is characterized as carcinogenic in humans [50]. An alternative to immunomodulary drugs and the side effects related to this could be radiation therapy, which shows promise in treating MUO [51].

When looking at the primary cause of death in the current population, the main cause of death or euthanasia was relapse (15/35 dogs, 42.9%), followed by a lack of response to treatment (9/35, 25.7%). It is important to keep in mind that the decision to euthanize is often complex, and several factors may affect the decision of the owner, as well as recommendations to euthanize by the responsible veterinarian. However, relapse in dogs suffering from MUO has also previously been described to correlate with a shorter survival time [22]. Preventing relapse and examining whether some treatment regimens have a lower relapse rate than others, would be of great interest to prolong survival. An important focus for future studies investigating different treatment regimens could potentially be the relapse rate.

Not surprisingly, the nature of the observed side effects was similar in all treatment groups and characteristic for CS, as CS was used in all treatment regimens. These included polyphagia (37/47 dogs, 78.7%), gastrointestinal signs (diarrhea in 29/47 dogs, 61.7%, vomiting in 17/47 dogs, 36.2%), and PU/PD (37/47 dogs, 78.7%). Due to the retrospective nature of this study, it was not possible to grade side effects objectively, nor to assess if side effects decreased as CS was tapered, or if tapering was done faster for the dogs receiving add-on drugs. A prospective study investigating possible correlations between CS dosage and side effects is warranted, as has been done for dogs suffering from SRMA [31]. Interestingly, in the present study only 2/63 dogs were euthanized as a direct result of side effects, but side effects might indirectly have led to euthanasia in two more dogs. Counting all four dogs as ‘death or euthanasia related to side effects’, they account for 6.3% of the total population, and 11.4% of the dogs that died during the study period, which should cause some concern.

Many unspecific side effects were reported during treatment with CS, and for some dogs, it may therefore be difficult to discriminate side effects from ordinary disease events that might have occurred anyway. For instance, corneal ulcers were seen in five dogs; however, they were all brachycephalic breeds (four pugs and one Chihuahua), which are known to have abnormal cranial conformation and buphthalmos, predisposing to corneal ulcers [52]. It is also worth noting that add-on drugs introduced to reduce side effects from CS-treatment have side effects of their own. For instance, vomiting, diarrhea, anorexia, weight loss, gingival hyperplasia, papillomatosis, hypertrichosis and excessive shedding have been described in a case report using Ci as monotherapy [53].

Due to its retrospective nature, this study has several limitations. Side effects were not recorded in a systematic manner in the medical records and may be underreported. In addition, recording the exact dosage at which specific side effects were seen was not done, and treatment protocols were not standardized, leading to different dosages and time of initiation of add-on drugs, as well as the timing of check-ups. Since data collection went back five years, a group of dogs were lost to follow-up despite attempts to contact owners, and clinical status for these dogs were therefore unknown.

The fact that dogs were recruited from two different hospitals allowed inclusion of a larger population; however, it also led to differences in methods of CSF analysis, and the use of different types of MRI and CT machines, as well as differences in preferred treatment protocols. We did also include dogs with a CT examination, but no MRI examination, which may have led to inclusion of dogs with an unclear diagnosis. Larger, multicentric studies might be necessary in order to include sufficient cases, but care should be taken to reduce the methodological differences between places.

limitation to this study was the lack of histopathological confirmation of the presumed diagnosis of MUO - however, as brain biopsies are rarely performed ante mortem, studies including only histologically confirmed MUO cases are usually strongly biased towards a short MST. However, we acknowledge the risk of misdiagnosis in the present study of dogs with presumed MUO, and that this may affect study data. For example, there is a risk that dogs with neoplastic lesions may have been included, affecting outcome negatively. Due to the lack of specific biomarkers for MUO, however, even prospective studies may be challenging in this matter.

When investigating treatment of MUO, a lack of knowledge of the underlying disease mechanisms poses a great challenge. Current treatment protocols aim at general immune suppression. Investigations of the underlying pathophysiology and identification of specific drug targets might aid in better treatment options with less side effects, as also suggested by Jeffery & Granger [54].

## Conclusion

The reported side effects to treatment were rarely a direct cause of death in the current study – however, side effects were reported for all dogs. Median survival time (MST) for dogs with MUO receiving corticosteroid monotherapy did not statistically differ from other treatment groups, but a possible bias towards more severe cases in the combination groups needs to be taken into account when interpreting results. Larger prospective studies, or, alternatively, retrospective studies, are needed to reach a conclusion.

Relapse was the number one cause of euthanasia, followed by a lack of response to treatment regardless of treatment group, indicating that the available treatment options are suboptimal. Clinical trials prospectively examining treatment alternatives are warranted if these can be conducted in an ethically acceptable manner. Further investigations into the underlying disease mechanisms are warranted.

## Data Availability

The dataset generated and analyzed during the current study are not publicly available due to information regarding specific dogs (owner information censored due to GDPR) and ethical approval has not been given for other use of collected data apart from the current study.

## List of abbreviations

CA: cytosine arabinoside
Ci: ciclosporin
CNS: central nervous system
CRI: constant rate infusion
CS: corticosteroids
CS + 2: corticosteroid therapy with at least two add-on drugs
CSF: cerebrospinal fluid
CT: computed tomography
FSAH: Fredrikstad Small Animal Hospital
GGT: gamma-glutamyl transferase
GME: granulomatous meningoencephalitis Le = leflunomide
Lo: lomustine
MMF: Mycophenolate Mofetil
MRI: magnetic resonance imaging
MUO: Meningoencephalitis of Unknown Origin
NLE: necrotizing leucoencephalitis
NME: necrotizing meningoencephalitis
P: *Probability*
PD: polydipsia
PU: polyuria
SRMA: steroid responsive meningitis arteritis
UHCA: University Hospital for Companion Animals
TNCC: total nucleated cell count
WBC: white blood cells
