# Correction to: Investigation of side effects to treatment and cause of death in 63 Scandinavian dogs suffering from meningoencephalitis of unknown origin: a retrospective study

**DOI:** 10.1186/s13028-024-00778-2

**Published:** 2024-10-28

**Authors:** Pernille Lindholm Heidemann, Bolette Erhald, Bodil Cathrine Koch, Hanne Gredal

**Affiliations:** 1 Department of Veterinary Clinical Sciences, University of Copenhagen, Dyrlaegevej 16, Frederiksberg, 1870 Denmark; 2 Evidensia Södra Djursjukhuset Kungens Kurva, Månskärsvägen 13, Kungens, Kurva 141 75 Sweden; 3 Fredrikstad Dyrehospital (Fredrikstad Small Animal Hospital), Wilbergjordet 2, Fredrikstad, 1605 Norway

The original version of this article [1] was revised: the body text contained incorrect parts. It was published as follows: Within the 5-year study period, 63 dogs were included. Of these, 35 (49.3%) died or were euthanized during the study period. Overall, 35/63 dogs (49.3%) died within the 5-year study period. Table 4 – All dogs – Number of deaths % − 49.3. Table 4 “Cause of death”.

It should be as follows: Within the 5-year study period, 63 dogs were included. Of these, 35 (55.6%) died or were euthanized during the study period. Overall, 35/63 dogs (55.6%) died within the 5-year study period. Table 5 – All dogs – Number of deaths % − 55.6. Table 5 “Cause of death”.
